# Replantation of Teeth

**Published:** 1880-02

**Authors:** 


### REPLANTATION OF TEETH.

The history of the replantation of teeth is so well known that we need not here reiterate it. But from the time of Hunter, who made known the possibility of transplanting teeth, and also wrote upon replanting teeth, these methods of treatment have been practiced by numerous individuals, both in Europe and America.

The question of replanting teeth appears to have had a somewhat spasmodic existence, and it has, notably during the present year, again been brought prominently before the profession.

In the number for March, 1876, of the *Bulletin et Memoires de la Société de Chirurgie*, there is a very long paper by Dr. Magitot on this subject, containing a report of fifty cases. An epitome of this article was read by Mr. Charles S. Tomes, before the Odontological Society of Great Britain in March last (see page 160 of the *Review*). In our February number, page 55, there is an article on replantation by Mr. George Torpey; and again in this issue, at page 521, there is published a very excellent lecture by Dr. Thompson.

From all that has been written on this subject we may set forth the following conclusions:

Where the periosteum is healthy, teeth may be extracted, pulp canals filled and replanted with a large per centage of successes.

Where the periosteum is in a state chronic inflammation, with the same treatment as before mentioned, there is a less percentage of successes.

In cases of alveolar abscess the best results are obtained when, in conjunction with replantation, a system of drainage is established.

It is in cases of alveolar abscess which are difficult to treat in the mouth, that the practice of replantation is most justifiable. To obtain drainage of the substances exuded during the healing process different methods have been adopted, such as a fistula through the alveolar process to the apex of the root, and to this may be added a seton; also by having a groove cut in the side of the root, from its apex to the neck of the tooth. But Dr. Thompson has devised a novel method of drainage by having a tube running through the centre of the root and opening upon the grinding surface of the tooth. When the cavity which was occupied by the abscess sac has healed up, and all exudation ceased, the tube can be accurately filled up by a pin, which was adjusted to the tube before replanting the tooth. This principle of drainage adopted by Dr. Thompson is the most complete and effectual, where it can be adopted, of any method hitherto made known; yet we must not overlook the position of a tooth so tubed in the lower jaw, in which case the discharge has to accumulate until it reaches and is taken up by the cotton wool dressing daily placed in the tube; whereas with such teeth in the upper jaw, gravitation favors the exudation quickly passing away. Nevertheless, the practical results, some of which we ourselves have witnessed, in all cases where this system of drainage has been adopted, have been eminently satisfactory.

There are numerous instances of replantation where alveolar abscess had existed, and no drainage had been provided, and the cases have done well. But there are evidently more failures, and less good results obtained, when alveolar abscess is thus treated without drainage, than when drainage is provided for.

When replanted teeth have become firm and useful, future trouble is not necessarily overcome; for in the course of one or more years the process of absorption may bring about the loss of the tooth. With the view of reducing the liability to absorption, Dr. Thompson excises the portion of the root denuded of periosteum, and restores this with a cap of gold, through which he also passes the drainage tube. It appears that this cap of gold at the apex of the root has been tolerated, indeed, has not given rise to any perceptible disturbance, for, so far, eight months. Though absorption of the gold is not at all likely, yet any portion of the tooth substance which is contained within the alveolus is liable to be so eaten away. The tendency to destructive absorption of the root may, perhaps, be lessened by the removal of the necrosed portion, which is generally considered as an intolerant irritant; but it remains for time and observation to teach us whether a foreign substance, such as a gold cap, in this situation is more acceptable to animate nature than the tissue of her deserted habitation.

The position we have attained with regard to replantation in cases of intractable alveolar abscess may, therefore, be said to be that this treatment, to insure the best results, should be in conjunction with drainage.—*Editorial in the Monthly Review of Dental Surgery.*

